# Advancements, Challenges, and Future Directions in Aquatic Life Criteria Research in China

**DOI:** 10.3390/toxics11100862

**Published:** 2023-10-16

**Authors:** Chen Liu, Zhaomei Geng, Jiayin Xu, Qingwei Li, Heng Zhang, Jinfen Pan

**Affiliations:** 1 Key Laboratory of Environment and Ecology (Ministry of Education), Ocean University of China, Qingdao 266100, Chinaxjy99929@gmail.com (J.X.); liqing-wei@outlook.com (Q.L.); ouczhangheng@outlook.com (H.Z.); 2 School of Mathematics, Sun Yat-Sen University, Guangzhou 510275, China; gengzhm@mail2.sysu.edu.cn; 3 Key Laboratory of Marine Eco-Environmental Science and Technology, First Institute of Oceanography, Ministry of Natural Resources, Qingdao 266061, China; 4 Laboratory for Marine Ecology and Environmental Science, Laoshan Laboratory, Qingdao 266200, China

**Keywords:** aquatic life criteria (ALC), water quality criteria (WQC), freshwater, priority pollutants, China

## Abstract

Aquatic life criteria (ALC) serve as the scientific foundation for establishing water quality standards, and in China, significant strides have been made in the development of freshwater ALC. This comprehensive review traces the evolution of China’s WQC, focusing on the methodological advancements and challenges in priority pollutants selection, test organism screening, and standardized ecotoxicity testing protocols. It also provides a critical evaluation of quality assurance measures, data validation techniques, and minimum data requirements essential for ALC assessments. The paper highlights China’s technical guidelines for deriving ALC, and reviews the published values for typical pollutants, assessing their impact on environmental quality standards. Emerging trends and future research avenues are discussed, including the incorporation of molecular toxicology data and the development of predictive models for pollutant toxicity. The review concludes by advocating for a tiered WQC system that accommodates China’s diverse ecological regions, thereby offering a robust scientific basis for enhanced water quality management.

## 1. Introduction

Water quality criteria (WQC) are essential for protecting aquatic ecosystems and human health. These criteria cover various areas such as aquatic life water quality criteria (ALC), human health water quality criteria, sediment quality criteria, and nutrient criteria [1].

The field of ALC research was first developed in the United States during the 1960s [2]. The U.S. later formalized this research by issuing comprehensive technical guidelines in 1985 [3], which have had a significant influence globally. In parallel, the European Union has made substantial advancements in aquatic risk assessment, with member states like the Netherlands contributing significantly [4]. Beyond the U.S. and EU, countries such as Canada [5], Australia, and New Zealand [6] have also conducted ALC research and developed their own technical guidelines.

When it comes to ALC formulation, the U.S. uniquely uses a dual-value system, incorporating both long-term and short-term ALC for each pollutant [3]. This approach was later adopted by Australia and New Zealand in their 2018 guideline updates [7]. While long-term ALC is used for daily water quality management, short-term ALC is designed to handle sudden water pollution incidents. Most other developed countries focus only on long-term ALC for daily management.

Developing ALC involves a complex process that includes careful screening of ecotoxicity data and choosing the right mathematical models for data analysis. The U.S. guidelines provide a comprehensive framework for this, covering aspects like toxicity endpoints, effect indices, exposure conditions, and data prioritization for both acute and chronic toxicity [3]. Different countries use different mathematical models; for example, the U.S. uses a log-triangle function model [3], the Netherlands uses a log-normal distribution model [4], and Australia and New Zealand use the Burr III model [6].

Quality assurance is crucial in toxicity data for developing reliable ALC. Developed countries have methods for assessing the quality of toxicity data, which can be either qualitative or quantitative. For instance, the U.S. [8] and the E.U. [9] use qualitative methods, while Australia and New Zealand use a quantitative approach [10]. These methods evaluate data quality based on various factors like the properties of the test substance, species characteristics, experimental design, exposure conditions, and statistical methods.

In terms of selecting test species, the U.S. guidelines recommend using native North American aquatic organisms [3]. Guidelines from other countries are less specific, lacking detailed recommendations or requirements about the geographical distribution of test species.

According to U.S. evaluation methods, toxicity data are categorized into quantitative data (used for environmental risk calculations), qualitative data (used to support environmental risk assessments), and invalid data. The E.U. method considers the reliability and relevance of the data and categorizes it into four types: unlimited reliable data, limited reliable data, unreliable data, and uncertain data. In Australia and New Zealand, toxicity data are scored and categorized into unacceptable, acceptable, and high-quality data based on these scores.

In China, ALC research has seen significant progress in recent years. This paper aims to provide a comprehensive overview of China’s ALC research, focusing on its historical development, priority pollutants and test species, data collection, technical guidelines, and published ALC values. This review is intended to serve as a valuable reference for the ongoing and future development of ALC. Despite the progress, several challenges continue to persist. These include the need for more expansive toxicity data, the development of reliable and standardized testing protocols, and the creation of a framework that can effectively translate scientific discoveries into actionable policies and standards.

## 2. The Evolution of Aquatic Life Water Quality Criteria in China

Research on water quality criteria (WQC) in China commenced in the 1980s, initially through the translation of American WQC Red Book and European WQC guidelines focused on fish protection. In the following years, some Chinese researchers conducted studies that utilized toxicity data from resident species in China. However, due to the lack of systematic research, China has largely relied on foreign WQC standards when establishing its own water quality guidelines. A notable example is the “China Surface Water Environmental Quality Standard” (GB 3838-2002), a cornerstone in China’s water management policies. This standard comprises 109 water quality criteria, the majority of which are adapted from international guidelines.

The turning point for WQC development in China came in 2005 following a significant water pollution incident involving nitrobenzene leakage in the Songhua River Basin. The emergency response adopted a nitrobenzene standard of 0.017 mg/L, which was based on U.S. criteria at the time. However, its applicability for protecting Chinese bodies of water remains a subject of debate. This incident catalyzed the advancement of WQC in China. The same year, the State Council of China set a national goal for “scientifically determining environmental criteria” in its “Decision on Implementing the Scientific Outlook on Development and Strengthening Environmental Protection.” During China’s Eleventh Five-Year Plan (2005–2010), several national projects were launched to support systematic WQC research [11].

In 2011, the Ministry of Science and Technology established the State Key Laboratory of Environmental Criteria and Risk Assessment, further boosting WQC research. In 2014, the revised “Environmental Protection Law” explicitly encouraged WQC research, marking the first legal recognition of WQC studies in China.

By 2017, the Ministry of Environmental Protection of China (MEPC) released the country’s inaugural batch of technical guidelines for WQC, covering freshwater ALC, human health water quality criteria, and lake nutrient criteria [12]. In 2018, the Ministry of Ecology and Environment of China (MEEC), formerly known as MEPC, included WQC development as part of its regular duties. In 2020, MEEC unveiled the first set of national ALC for substances like cadmium [13], ammonia nitrogen [14], and phenol [15], signifying a landmark achievement in China’s ALC research (Table 1).

## 3. Methodological Approaches for Priority Pollutants Screening in ALC Studies

**Figure 1:**
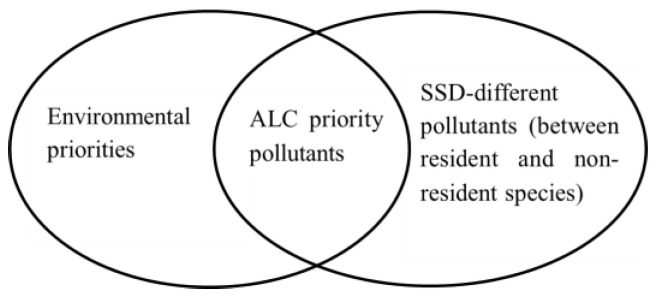
Principles for ALC priority pollutants screening.

Given the labor-intensive and time-consuming nature of environmental criteria research, and considering the multitude of both individual and grouped pollutants in the environment, prioritization is imperative. It is vital to identify not only individual pollutants that pose significant risks, but also to acknowledge and prioritize groups of substances with similar purposes and effects, such as pesticides or PFAS compounds. This nuanced approach ensures comprehensive coverage, addressing both individual pollutants and categories of substances warranting immediate attention, thereby facilitating more effective and encompassing environmental protection strategies. While the topic of priority pollutants screening in water environments is widely discussed, the criteria for selecting priority pollutants for ALC research are diverse (Figure 1). Two key conditions must be met: first, the pollutant should be of concern in water management; second, there should be a significant difference in species sensitivity distribution (SSD) between resident and non-resident species. This ensures that the derived criteria values differ substantially depending on whether resident or non-resident species data are used. If no such SSD difference exists, national water quality standards can be temporarily based on foreign criteria, and the pollutant is not considered a priority for ALC research.

Yan et al. [16] conducted a comprehensive study targeting 160 priority pollutants identified by the U.S., the E.U., and China. They collected and analyzed acute toxicity data for these pollutants in freshwater aquatic organisms. Their findings revealed that the HC_5_ values (Hazardous Concentration affecting 5% of species, a key metric in ALC derivation) for certain pollutants varied significantly. As a result, 24 pollutants across six categories were identified as priority pollutants for ALC research in China (Table 2). Pesticide compounds were most prevalent, followed by metals and phenols. This distribution is also influenced by the availability of ecotoxicity data; many pollutants could not be adequately assessed due to insufficient data. As more toxicity data become available, it is likely that additional pollutants will be classified as priority pollutants for ALC research.

Currently, a significant challenge is the scarcity of toxicity data for a broad spectrum of pollutants. This limitation obstructs the process of identifying priority pollutants for ALC research in China. Furthermore, the absence of systematic research and dependence on international WQC standards complicate the development of criteria that are meticulously designed for the distinctive biodiversity and aquatic ecosystems present in China.

## 4. Criteria for the Selection of Test Organisms in Aquatic Ecotoxicology

The biodiversity of aquatic ecosystems varies significantly across different countries, thereby influencing the target organisms for aquatic life criteria (ALC). Identifying species that are particularly sensitive to pollutants is crucial for the development of accurate ALC. While water quality criteria (WQC) studies have generally lacked a systematic approach to selecting sensitive aquatic organisms, the U.S. ALC guidelines [3] provide a list of recommended North American aquatic species. However, the sensitivity of these listed species has not been rigorously evaluated.

Yan et al. [17] developed a method for screening ALC test organisms based on the distribution characteristics of freshwater species in China. Utilizing species sensitivity analyses, they systematically identified sensitive aquatic organisms across various categories, including amphibians [18], fish [19], crustaceans [20], aquatic insects [21], mollusks [22], annelids [23], and aquatic plants [12]. In total, 46 sensitive freshwater species spanning seven phyla were identified. These include three species of coelenterates, one species of flatworms, three species of rotifers, two species of annelids, three species of mollusks, 13 species of arthropods, 11 species of chordates, three species of green algae, one species of diatoms, one species of ferns, and five species of angiosperms. These species have been recommended as test organisms for China’s ALC research and are detailed in the supplementary materials (Table S1) of the Chinese ALC guidelines [12].

## 5. Standardized Ecotoxicity Testing Protocols

The development of standardized ecotoxicity testing methods is foundational for generating reliable ecotoxicity data. Currently, China has established a range of national standard methods for ecotoxicity testing, encompassing both acute and chronic toxicity tests for fish, chironomids, daphnia, and algae (Table 3). However, for other freshwater organisms like shellfish, annelids, and rotifers, China has yet to establish standard testing protocols. In these cases, researchers rely on international standards or methods published in scientific literature for ALC studies.

Given that ALC development requires extensive toxicity data, including data from non-standard test organisms, there is an urgent need to develop additional testing methods. Existing Chinese standards do not yet cover the full spectrum of freshwater biological groups. To address this gap, Chinese researchers are in the process of developing standard test methods for rotifers, water worms, mollusks, planaria, and region-specific fish species. In the interim, non-standard test methods continue to be employed for toxicity testing in ALC research. The lack of standardized testing protocols for a variety of freshwater organisms poses a significant challenge. This gap forces researchers to depend on international standards or methods documented in scientific literature. However, these might not always be applicable or reflective of the rich diversity of aquatic life in China.

## 6. Quality Assurance and Data Validation in Aquatic Ecotoxicological Studies

Ensuring the quality of toxicity data is fundamental for the development of robust water quality standards. As early as the 1990s, Klimisch et al. [24] introduced a method for assessing the quality of toxicity data. Subsequent studies [25,26,27] have expanded on this, although the reliability of their evaluation outcomes has been questioned [28].

In 2011, the U.S. Environmental Protection Agency released guidelines specifically aimed at quality assessment in ALC-related ecotoxicity studies. These guidelines provide a qualitative framework for toxicity data assessment, covering aspects such as data screening, evaluation, classification, and application [29]. Similarly, the European Union has established the Criteria for Reporting and Evaluating Ecotoxicity Data (CRED), which assesses data quality based on its reliability and relevance [9]. Australia and New Zealand followed suit, issuing their own guidelines for ecotoxicity data assessment in 2018 [30].

Drawing upon methodologies from Western countries, Chinese researchers have proposed a quantitative approach for evaluating the quality of ecotoxicity data. This approach considers five key aspects: data sources, chemical reagents, test organisms, experimental procedures, and experimental outcomes. Based on the evaluation scores, toxicity data are categorized into three levels: high-quality, acceptable, and unacceptable for ALC development in China. These categories are further detailed in the Supplementary Materials (Table S2).

## 7. Minimum Data Requirements and Data Prioritization Strategies for ALC Development

### 7.1. Minimum Toxicity Data Requirements (MTDR)

MTDR serve as a cornerstone for deriving ALC values. Developed countries have distinct MTDR frameworks; for example, the U.S. guidelines mandate data from eight families of aquatic animals and one aquatic plant [3], whereas other nations require data from five or six families [4,5]. In China, scholars have tailored MTDR to the nation’s nascent ALC development stage. According to China’s ALC guideline (HJ 831-2022), the MTDR encompasses data from five aquatic animals—specifically, one Cyprinidae fish, one non-Cyprinidae teleost fish, one zooplankton, one mollusk or benthic crustacean, and one amphibian or another phylum of animals—as well as one aquatic plant. Furthermore, toxicity data for a minimum of 10 species must be collected to derive the water quality criteria (WQC). As the volume of ecotoxicity data for native Chinese species grows, these MTDR are expected to evolve accordingly.

### 7.2. Data Prioritization Strategies

Both acute and chronic toxicity data are essential for dual-value ALC studies. These data come in various forms, with chronic toxicity indices including no observed effect concentration (NOEC), lowest observed effect concentration (LOEC), maximum acceptable toxic concentration (MATC), lethal concentration of 50% tested species (LC_50_), and concentration for x% of maximal effect (EC_X_), among others. Factors such as the life stage of the test organism, the taxonomic category of the data, the exposure methodology, and the monitoring of pollutant concentrations can all influence toxicity test outcomes. Consequently, establishing data prioritization is crucial in WQC studies. National requirements on this issue vary; for instance, the U.S. prioritizes genus-level toxicity data and favors the use of MATC [3], while most other countries prioritize NOEC for long-term WQC derivation [5,7,31]. Comparative studies have also been conducted to analyze the relationship between EC_10_ and NOEC [32]. In the updated 2022 China Freshwater Biological Water Quality Criteria Guidelines (HJ 831-2022), the prioritization hierarchy for chronic toxicity indices is as follows: MATC > EC_20_ > EC_10_ = NOEC > LOEC > EC_50_ > LC_50_. Additionally, data from sensitive life stages, monitored pollutant concentrations, and flow toxicity experiments are given precedence in ALC derivation.

## 8. Technical Guidelines for the Development and Implementation of ALC in China

China’s inaugural technical guideline for freshwater ALC was released in 2017, adopting a dual-value system comprising both long-term and short-term ALC [33]. The guideline outlines a structured approach to ALC development, encompassing phases such as target pollutant identification, data collection and screening, ALC derivation, and technical report compilation. It specifies that test species should be those commonly found in various freshwater ecosystems across China. Data for acute and chronic ecotoxicity of target pollutants are sourced from databases like Web of Science, as well as domestic and international toxicity databases like ECOTOX, and are screened based on stringent criteria.

Four statistical models—normal, log-normal, logistic, and log-logistic—are employed to fit the species sensitivity distribution (SSD) curve. The optimal model is selected based on fitting parameter comparisons. The HC_5_ value, fundamental for ALC calculation, is then derived using an optimal model and adjusted with a correction factor to reduce uncertainties in real-world conditions. The factor applied depends on the available toxicity data; a factor of two for 15 species, and a factor of three for 10 to 14 species ensures accurate and relevant ALC calculations for China’s specific environmental contexts. Acute data inform the short-term ALC, while chronic data are used for the long-term ALC.

In 2020, following these guidelines, the Ministry of Ecology and Environment of China (MEEC) issued national ALC documents for cadmium [13], ammonia nitrogen [14], and phenol [15]. In 2022, the 2017 guidelines underwent a comprehensive revision, culminating in the release of the updated version (HJ 831-2022). This revised edition incorporates various modifications, including changes in criteria derivation methods, the details of which are elaborated on in a published paper [34].

## 9. A Review of Published ALC Values for Pollutants in Chinese Aquatic Ecosystems

Over a decade of accelerated research has yielded published ALC values for a range of key pollutants in China, including ammonia nitrogen, metals, pesticides, endocrine disruptors, and emerging contaminants (Table 4). These values serve as valuable criteria for updating China’s surface water quality standards.

In 2020, the Ministry of Ecology and Environment of China (MEEC) officially unveiled national ALC values for cadmium and ammonia nitrogen, marking a significant milestone in China’s ALC research landscape.

As China contemplates updates to its surface water quality standards, these published ALC values are poised to make a constructive contribution to the revision process.

## 10. Future Directions and Emerging Trends in Aquatic Life Water Quality Criteria

### 10.1. A Milestone in Chinese ALC Research

China has made significant strides in establishing its own ALC technical methodology and publishing national criteria. This progress underscores the remarkable advancements in ALC research within the country. Chinese scholars are actively exploring various facets to further refine the WQC methodology, thereby providing a more robust scientific foundation for future developments.

### 10.2. Innovations in Methodology

Traditionally, international ALC methodologies have relied on individual-level toxicity data. However, Yang et al. [64] have pioneered a new approach that incorporates molecular toxicology and community-level data. Specifically, they developed an ecological threshold for ammonia nitrogen in Lake Tai based on the response of the lake’s phytoplankton community to ammonia concentration changes. As molecular toxicological data continue to grow, researchers are investigating how to integrate this information into ALC development [65].

### 10.3. Predictive Modeling

Chinese scholars have also focused on predictive modeling to estimate pollutant toxicity. Various models have been developed, including those for heavy metal ecotoxicity [66], endocrine-disrupting compound (EDC) reproductive toxicity [67,68], pesticide ecotoxicity [69], and BTEX substances [70]. These efforts contribute to the enrichment of native Chinese ecotoxicity data and the refinement of the country’s ALC methodology.

### 10.4. Bridging the Gap between WQC and Legal Standards

In China, WQC are viewed as scientifically-derived safety thresholds without legal force, while water quality standards are legally binding and consider economic, technical, and management factors. The challenge lies in translating WQC into actionable water quality standards. Currently, emergency standards, which do not factor in economic costs, are easier to establish. However, creating regular standards remains complex, and no universally accepted approach has been proposed yet.

Given China’s vast geographical diversity, there is active exploration into establishing a tiered WQC system, such as a “state-basin-region” ALC system. This would support more nuanced and region-specific water management strategies across China’s various basins.

## Figures and Tables

**Table 1:** Landmark events in the development of ALC in China.

Year	Events	Related Ministries
2005	National goal for “scientifically determining environmental criteria” set	State Council of China
2011	State Key Laboratory of Environmental Criteria and Risk Assessment established	Ministry of Science and Technology of China
2014	Encouragement of WQC research included in the revised Environmental Protection Law	National People’s Congress of China
2017	First batch of technical guidelines for WQC issued	MEPC
2018	WQC development incorporated into MEEC duties	State Commission of Public Sectors Reform
2020	First batch of national ALC was released	MEEC
2022	First technical guidelines for marine organism protection issued	MEEC

**Table 2:** Chinese ALC priority pollutants [16].

No.	CAS Number	Pollutants	Classification
1	7440-41-7	Be(II)	Metal
2	7440-43-9	Cd(II)	Metal
3	7440-47-3	Cr(VI)	Metal
4	7440-02-0	Ni(I)	Metal
5	57-74-9	Chlordane	Pesticide
6	60-57-1	Dieldrin	Pesticide
7	115-29-7	Endosulfan	Pesticide
8	72-20-8	Endrin	Pesticide
9	76-44-8	Heptachlor	Pesticide
10	608-73-1	Hexachlorocyclohexane	Pesticide
11	309-00-2	Aldrin	Pesticide
12	8001-35-2	Toxaphene	Pesticide
13	60-51-5	Dimethoate	Pesticide
14	298-00-0	Parathion-methyl	Pesticide
15	52-68-6	Trichlorfon	Pesticide
16	1912-24-9	Atrazine	Pesticide
17	470-90-6	Chlorfenvinfos	Pesticide
18	1582-09-8	Trifluralin	Pesticide
19	108-92-2	Phenol	Phenol
20	120-83-2	2, 4–Dichlorophenol	Phenol
21	51-28-5	2, 4–Dinitrophenol	Phenol
22	206-44-0	Fluoranthene	PAHs
23	/	Tributyltin compounds	Organotin
24	7664-41-7	Ammonia	Common chemical

**Table 3:** China national standard toxicity test guidelines for freshwater organisms.

Species Group	Test Guideline	Guideline Number
Fish	Water quality—Determination of the acute toxicity of substances to a freshwater fish (*Brachydanio rerio* Hamilton-Buchanan)	GB/T 13267-1991
Fish	Chemicals—Fish acute toxicity test	GB/T 27861-2011
Fish	Chemicals—Fish (*Oryzias latipes*, d-rR medaka) early life stage toxicity test	GBT 29764-2013
Fish	Chemicals—Fish, juvenile growth test	GB/T 21806-2008
Fish	Testing of chemicals—Fish, short-term toxicity test on embryo and sac-fry stages	GB/T 21807-2008
Fish	Chemicals—Fish, early-life stage toxicity test	GB/T 21854-2008
Fish	Chemicals—Rare minnow (*Gobiocypris rarus*) acute toxicity test	GB/T 29763-2013
Daphnia	Method for acute toxicity test of *Daphnia magna* straus	GB/T 16125-2012
Daphnia	Chemicals—*Daphnia magna* reproduction test	GB/T 21828-2008
Chironomid	Chemicals—Sediment-water chironomid toxicity test—Spiked water method	GB/T 27858-2011
Chironomid	Chemicals—Sediment-water chironomid toxicity test—Spiked sediment method	GB/T 27859-2011
Alga	Chemicals—Algae growth inhibition test	GB/T 21805-2008
Duckweed	Chemicals—*Lemna* sp. growth inhibition test	GB/T 35524-2017

**Table 4:** Published ALC values in China.

Chemicals		Short-term ALC (μg/L)	Long-term ALC (μg/L)	References
Ammonia nitrogen		12,000 (20 °C, pH 7.0) (National criteria)	1500 (20 °C, pH 7.0) (National criteria)	[14,35]
Cd(II)		4.2 (hardness = 100 mg/L) (National criteria)	0.23 (hardness = 100 mg/L) (National criteria)	[13]
Zn(II)		48.43	20.01	[36]
Zn(II)		230.6	/	[37]
Pb(II)		90.7 (hardness = 100 mg/L)	2.1 (hardness = 100 mg/L)	[38]
Cr(VI)		45.79	14.22	[36]
Cu(II)		1.391	0.495	[39]
		/	0.87–1.49	[40]
Ag(I)		e^1.58lnH − 8.68^ *	e^1.58lnH − 10.28^ *	[41]
As	As(III)	167	42	[42]
	As(V)	384	44	
Chloride		/	187,500	[43]
Phenol		2472	316.2	[44]
Benzene		2651	530.2	[36]
Nitrobenzene		18	1	[45]
Phenanthrene		51.4	18.6	[46]
PAEs		/	0.04–41.9	[47]
Pentachlorophenol		13.21 (pH = 7.8)	1.20 (pH = 7.8)	[48]
Atrazine		/	0.044	[49]
2,4-dichlorophenol		1250	212	[50]
		/	9–44	[51]
2,4,6-trichlorophenol		1010	226	[52]
		/	57	[53]
Dichlorvos		1.33	0.132	[54]
Glyphosate		3350	260	[55]
Malathion		0.100	0.008	[54]
DEET		21,530	520	[56]
Triphenyltin		0.396 (Sn)	0.0056 (Sn)	[57]
PFOS		3780	250	[58]
PFOA		45,540	3520	[58]
Triclosan		9	2	[59]
TBBPA		147.5	12.6	[60]
HBCD		2320	128	[61]
PBDEs		49.2–239	10.3–26.7	[62]
TDCPP		877 (HC_5_)	0.03333 (HC_5_)	[63]

* H: hardness of water.

## Data Availability

Not applicable.

## Supplementary Materials

The following supporting information can be downloaded at: https://www.mdpi.com/article/10.3390/toxics11100862/s1, Table S1. Recommended Chinese resident freshwater test organisms for the development of ALC; Table S2. Evaluation criteria for toxicity data in China’s aquatic life criteria (ALC) development.
