# Supplementary material for: Advancements, Challenges, and Future Directions in Aquatic Life Criteria Research in China
Source: Toxics. 2023 Oct 16;11(10):862. doi: 10.3390/toxics11100862 (PMC10667990; doi:10.3390/toxics11100862)
Supplement: Supplementary file 1 [file toxics-11-00862-s001.zip › toxics-2630907-supplementary.pdf]

Advancements, Challenges, and Future Directions in Aquatic Life Criteria Research in China

Chen Liu [1], Zhaomei Geng [2], Jia-yin Xu [1,3], Qing-Wei Li [1,3], Heng Zhang [1,3] and Jin-Fen Pan [1,4]*

1 Key Laboratory of Environment and Ecology (Ministry of Education), Ocean University of China, Qingdao 266100, China

2 School of Mathematics, Sun Yat-Sen University, Guangzhou 510275, China

3 Key Laboratory of Marine Eco-Environmental Science and Technology, First Institute of Oceanography, Ministry of Natural Resources, Qingdao 266061, China

4 Laboratory for Marine Ecology and Environmental Science, Laoshan Laboratory, Qingdao 266200, China

*Corresponding author: jfpan@ouc.edu.cn (J.-F.P.)

Supplementary materials

Two Tables:

Table S1: Recommended Chinese resident freshwater test organisms for the development of ALC.
Table S2: Evaluation criteria for toxicity data in China's aquatic life criteria (ALC) development.

Table S1 Recommended Chinese resident freshwater test organisms for the development of ALC.

No.	Scientific name	Taxonomy	
1	*Hydra oligactis*	Cnidaria	Hydridae
2	*Hydra viridis*	Cnidaria	Hydridae
3	*Hydra vulgaris*	Cnidaria	Hydridae
4	*Dugesia japonica*	Platyhelminthes	Tricornutricidae
5	*Brachionus calyciflorus*	Rotifera	Brachiopodiaceae
6	*Keratella cochlearis*	Rotifera	Brachiopodiaceae
7	*Lecane quadridentata*	Rotifera	Coelomonaceae
8	*Branchiura sowerbyi*	Annelida	Tubificidae
9	*Tubifex tubifex*	Annelida	Tubificidae
10	*Semisulcospira libertine*	Mollusca	Thiaridae
11	*Lymnaea stagnalis*	Mollusca	Spirulinae
12	*Corbicula fluminea*	Mollusca	Corbiculidae
13	*Daphnia magna*	Arthropoda	Daphniidae
14	*Daphnia pulex*	Arthropoda	Daphniidae
15	*Daphnia cucullata*	Arthropoda	Daphniidae
16	*Daphnia hyaline*	Arthropoda	Daphniidae
17	*Simocephalus serrulatus*	Arthropoda	Daphniidae
18	*Ceriodaphnia dubia*	Arthropoda	Daphniidae
19	*Gammarus pulex*	Arthropoda	Gammaridae
20	*Gammarus lacustrid*	Arthropoda	Gammaridae
21	*Macrobrachium nipponnense*	Arthropoda	Palaemonidae
22	*Eriocheir sinensis*	Arthropoda	Grapsidae
23	*Baetis Rhodani*	Arthropoda	Ephemeraceae
24	*Heptagenia sulphurea*	Arthropoda	Heptageniidae
25	*Contaminated Brachythemis*	Arthropoda	Libellulidae
26	*Cyprinus carpio*	Chordate	Cyprinidae
27	*Ctenopharyngodon idellus*	Chordate	Cyprinidae
28	*Hypophthalmichthys molitrix*	Chordate	Cyprinidae
29	*Aristichthys nobilis*	Chordate	Cyprinidae
30	*Carassius auratus*	Chordate	Cyprinidae
31	*Pseudorasbora parva*	Chordate	Cyprinidae
32	*Misgurnus anguillicaudatus*	Chordate	Cobitidae
33	*Pelteobagrus Fulvidraco*	Chordate	Coelomeraceae
34	*Monopterus albus*	Chordate	Synbranchiidae
35	*Siniperca chuatsi*	Chordate	Percichthyidae
36	*Spiny Quasipaa*	Chordate	Ranidae

37	*Chlamydomonas reinhardtii*	Chlorophyta	Chlamydomonadacea
38	*Pseudokirchneriella subcapitata*	Chlorophyta	Chlorella
39	*Scenedesmus acutus*	Chlorophyta	Scenedesmaceae
40	*Navicula pelliculosa*	Bacillariophyta	Naviculaceae
41	*Salvinia natans*	Pteridophyta	Robiniaceae
42	*Lemna minor*	Angiosperms	Lemnaceae
43	*Spirodela polyrrhiza*	Angiosperms	Lemnaceae
44	*Potamogeton crispus*	Angiosperms	Potamogetonaceae
45	*Hydrilla verticillata*	Angiosperms	Hydrocharitaceae
46	*Ceratophyllum demersum*	Angiosperms	Ceratophyllaceae

Table S2 Evaluation criteria for toxicity data in China's aquatic life criteria (ALC) development.

Item No.	Evaluation criteria	Scoring options (answers and scores*)
Data sources		
1	Do the data come from regular papers or authoritative reports?	Yes (2), No (Invalid)
2	Is the full-text paper or report available?	Yes (2), No (Invalid)
3	Are the toxicity data on an individual level?	Yes (2), No (Invalid)
4	Did the toxicity data come from a resident species?	Yes (2), No (Invalid)
Test substances		
5	Did the test substances have a definite name and appropriate chemical form?	Yes (3), No (0)
6	Were the test substances of an analytical grade or of the highest purity available?	Yes (3), No (0)
Test organisms		
7	Were the characteristics of the test organism reported (such as body length, body weight, age)?	Yes (4), No (0)
8	Was the test organism free from pre-exposure to the test substance?	Yes (4), No (Invalid)
9	Was the non-lethal performance of the control organism normal?	Yes (4), No (0)
Experimental process		
10	Has the contamination of the control group been tested?	Yes (3), No (0), The control group species was polluted (Invalid)
11	Did the test process meet the test method standards?	Yes (4), No (0)
12	Was a suitable control group designed (blank control and cosolvent control, etc.)?	Yes (4), No (Invalid)
13	Did the control and test groups contain duplicates?	Yes (4), No (0)
14	Did the test medium meet the requirements (freshwater, seawater, etc.)?	Yes (4), No (Invalid)
15	Did the literature report the type of exposure (static, flow-through, etc.)?	Yes (4), No (0)
16	Did the test organism have an appropriate exposure time?	Yes (8), No (0)
17	Did the experiment have appropriate acute or chronic toxicological endpoints?	Yes (10), No (Invalid)
18	Was the chemical concentration of the solution measured?	Yes (4), No (0)
19	If necessary, has a reference substance toxicity test been conducted?	Yes (4), No (0)
20	Was the ratio between the concentration of the test solution appropriate?	Yes (4), No (0)
21	Was the load of the test organism in the test system appropriate?	Yes (4), No (0)
Experimental results		
22	Was the dose-response relationship expressed?	Yes (4), No (0)

23	Was the correct statistical method used to obtain the results?	Yes (4), No (0)
24	For the lethal concentration (LC) or effect concentration (EC), was a confidence interval provided? For the no observed effect concentration (NOEC) or lowest observed effect concentration (LOEC), was it statistically significant?	Yes (4), No (0)
25	Was the monitoring of water quality parameters that may affect the toxicity of pollutants adequate?	Yes (5), Basically yes (3), No (Invalid)
Total score		100

*The results of "invalid" indicate the data cannot be accepted to be used for deriving WQC.

Data Classification:

Total score ≥ 80: High-quality data

80 > Total score ≥ 60: Acceptable data

60 > Total score: Unacceptable data
